# Caution to psychiatry ward: COVID‐19 pneumonia can manifest weeks or months after testing positive with a PCR test in individuals on preexisting immune‐suppressing medication

**DOI:** 10.1002/pcn5.135

**Published:** 2023-08-27

**Authors:** Masaki Nakano, Michitaka Funayama, Riko Wakisaka, Taketo Takata, Shun Kudo, Shin Kuramochi, Akihiro Koreki, Satoyuki Ogino, Takuto Ishida, Hiroyuki Uchida, Masaru Mimura

**Affiliations:** ^1^ Department of Neuropsychiatry Ashikaga Red Cross Hospital Ashikaga Tochigi Japan; ^2^ Department of Neuropsychiatry Keio University School of Medicine Shinjuku Tokyo Japan; ^3^ Department of Emergency and Critical Care Medicine Nippon Medical School Tokyo Japan; ^4^ Department of Neuropsychiatry Kawasaki Municipal Hospital Kawaski Kanagawa Japan; ^5^ Department of Psychiatry National Hospital Organization Shimofusa Psychiatric Medical Center Chiba Japan; ^6^ Department of Trauma and Critical Care Medicine Kyorin University School of Medicine Mitaka Tokyo Japan; ^7^ Tokyo Metropolitan Matsuzawa Hospital Tokyo Japan

**Keywords:** COVID‐19, pneumonia, psychiatry ward, immune‐suppressing medication

## Abstract

**Background:**

Some patients are reported to develop depression immediately after COVID‐19 infection. Typically, hospitalization is arranged a week to 10 days after symptom onset to avoid outbreak in the psychiatric ward when infectivity is almost eliminated. However, in patients on immunosuppressive drugs, infection is known to persist beyond the 10th day after testing positive with a polymerase chain reaction (PCR) test.

**Case Presentation:**

We present a patient with follicular lymphoma who was receiving immune‐suppressing medication and contracted a COVID‐19 infection; she developed severe depression and eventually required hospitalization 10 days after symptom onset or 5 days after the COVID‐19 infection‐related symptoms disappeared. Although the patient did not exhibit any symptom of pneumonia upon admission, she developed COVID‐19 pneumonia 3 weeks after the initial positive test. She received intravenous infusion of the antiviral drug remdesivir, which led to the improvement of pneumonia, and she was discharged on day 32 from testing COVID‐19 positive. However, COVID‐19 pneumonia recurred on days 64 and 74.

**Conclusion:**

This is the first report of COVID‐19 pneumonia developing in a psychiatric ward in a patient on immunosuppressive drugs, weeks to months after testing positive with a PCR test. When patients with compromised immune function, such as those on immunosuppressant medication or those with human immunodeficiency virus disease, are admitted to a psychiatric ward, careful monitoring of the risk of recurrence and sufficient consideration for infection control measures are necessary to avoid outbreaks.

## BACKGROUND

Psychiatry wards are exposed to a higher risk of COVID‐19 outbreaks due to difficulties in controlling the spread of infection.^1^, ^2^, ^3^ As part of infection control strategies, mandatory screening tests, such as polymerase chain reaction test (PCR test), and universal mask‐wearing have been suggested.^4^ On the other hand, the implementation of those control strategies has recently decreased due to milder symptoms associated with the omicron variant. Still, outbreaks of COVID‐19 in psychiatric wards continue to pose significant risks for vulnerable population groups, such as elderly patients.

For mild to moderate cases of COVID‐19, the presence of infectious virus is highly unlikely beyond the 10th day of symptom onset.^5^ Alternatively, the infectivity may decrease after 3 days of symptom improvement following COVID‐19 infection.^6^ These facts are taken into consideration when determining the optimal timing for admission of such patients. This issue is critically important since some COVID‐19 patients require psychiatric hospitalization in the light of a surge in the occurrence of depression immediately following the infection.^7^

In this report, we present a patient with follicular lymphoma who was receiving immune‐suppressing medication and contracted a COVID‐19 infection; she developed severe depression and eventually required hospitalization 10 days after symptom onset or 5 days after the COVID‐19 infection‐related symptoms had disappeared. Although the patient did not exhibit any symptom of pneumonia upon admission, she developed COVID‐19 pneumonia 3 weeks after the initial positive test. Such cases may not be so uncommon, even in psychiatric wards, because it is known that patients undergoing drug therapy for lymphoma have more severe depressive symptoms than patients who are not undergoing drug therapy for lymphoma.^8^

## CASE PRESENTATION

The patient was a 76‐year‐old woman who used to work at a self‐owned shop. In her 40s, she had experienced dizziness and vomiting, which led to a diagnosis of autonomic nervous system dysfunction. This condition eventually resolved naturally. After her husband's sudden death from cancer at the age of 74, she had occasionally experienced episodes of anxiety, which did not require any treatment. During that period, she developed follicular lymphoma and was treated with bimonthly infusions of 1000 mg of obinutuzumab (a molecular‐targeted medication with long‐term immunosuppressive side‐effects due to its direct destruction of B cells) for a period of 18 months. Her last dose was administered 29 days before she contracted COVID‐19.

At the age of 76 years, the patient had developed a cough and tested positive for COVID‐19 by PCR test (day 0). Considering the timing of onset in the latter half of 2022, she was presumed to have been infected with the omicron variant. She was treated with molnupiravir, an antiviral drug for COVID‐19, but experienced nausea and discontinued the drug 3 days later. Despite the resolution of coughs on day 6, she continued to experience persistent nausea and became restless. Her symptoms led to a visit to the emergency department on day 8. Blood tests and her head computed tomography (CT) scan showed no abnormalities. On day 10, she exhibited severe restlessness, a substantial loss of appetite, severe suicidal ideation, and a depressive mood. According to the DSM‐5 criteria, she was diagnosed with major depression with a score of 52 on the Montgomery–Åsberg Depression Rating Scale (MADRS) indicating severe depression.^9^ She was admitted to our psychiatry ward on day 11. Since her COVID‐19 symptoms had already disappeared and the recent infection could result in a positive finding in reverse transcriptase loop‐mediated isothermal amplification test (RT‐LAMP test), no screening test was performed. Blood tests on admission showed normal inflammatory markers with minimal evidence of infection. Likewise, her chest CT scan on admission showed no abnormal findings. She then underwent modified electroconvulsive therapy (mECT). Infection‐prevention measures were taken during hospitalization and the mECT procedure. After four sessions of mECT, her depressive symptoms were resolved.

**Figure 1:**
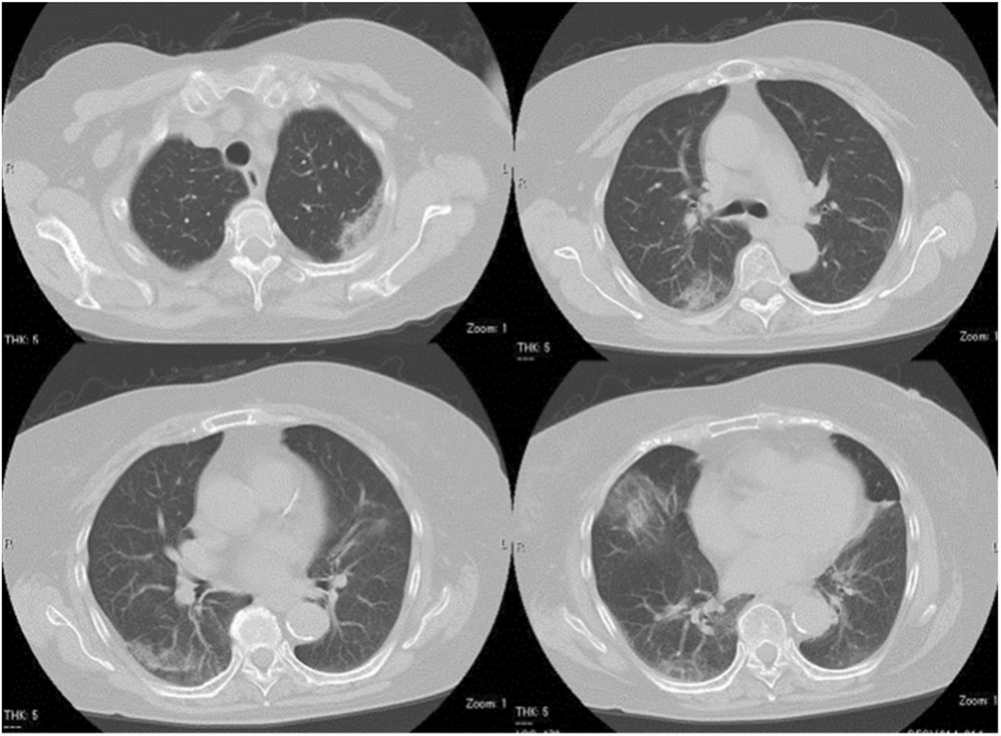
Chest computed tomography scan taken 3 weeks after the initial positive test. The images indicate the presence of scattered ground glass and reticular opacities in the peripheral areas of both lungs, suggesting the development of COVID‐19 pneumonia.

On day 21, however, she presented with a fever of 38°C and a cough, leading to further investigations. The result of her RT‐LAMP test was positive, and her chest CT scan revealed scattered ground glass and reticular opacities in the peripheral areas of both lungs (Figure 1), leading to a diagnosis of COVID‐19 pneumonia. We monitored the blood lymphocyte count closely due to obinutuzumab's action as an anti‐CD20 monoclonal antibody, which directly destroys B cells and may lead to lymphopenia. The lymphocyte count had dropped to 700/μL on day 21, although it had been 1900/μL on day 8. This decrease in lymphocyte count continued to progress gradually over a long period of time, reaching 200/μL on day 94. On the other hand, the neutrophil count was 2500/μL on day 21 and remained above 2500/μL without any decline thereafter. From day 21, the patient received intravenous infusion of the antiviral drug remdesivir along with a short period of oxygen therapy, which led to the improvement of pneumonia, and she was discharged on day 32. However, COVID‐19 pneumonia recurred on days 64 and 74. No COVID‐19 outbreaks were recorded in the psychiatric ward during her hospitalization.

## DISCUSSION

This is the first report of COVID‐19 pneumonia developing in a psychiatric ward in a patient on immunosuppressive drugs, weeks to months after testing positive with a PCR test. In immunocompromised patients and those receiving immune‐suppressing medication, there have been reports of prolonged and/or severe COVID‐19 infection.^10^, ^11^ In this case, the patient initially exhibited only mild symptoms, such as cough, when testing positive for COVID‐19 but developed pneumonia at a delayed timing of day 21. The delayed onset of COVID‐19 pneumonia may have been due to the depletion of B cells and reduced humoral immunity caused by obinutuzumab administration, which suppressed the immune response during COVID‐19 infection, resulting in the delayed appearance of clinical symptoms. There have been several reports of delayed onset of COVID‐19 pneumonia in cases where the same anti‐CD20 monoclonal antibody, rituximab, was administered.^12^ This delayed response is comparable to that seen in immune reconstitution inflammatory syndrome, in which the immune response to pathogens becomes apparent as the immune system is restored.^12^ In fact, during the development of COVID‐19 pneumonia, the number of lymphocytes decreased significantly from 1900/μL on day 8 to 700/μL on day 21. This decline reflects a late immune response and lymphocyte trafficking, where lymphocytes in the peripheral blood migrate to the lungs. Such a phenomenon is often observed during viral infections with severe symptoms, including COVID‐19.^13^ The continuous decline in the number of lymphocytes down to 200/μL indicates lymphopenia, defined as a lymphocyte count of less than 1000/μL.^14^ In this patient, lymphopenia is primarily caused by the depletion of B cells and, to a lesser extent, T lymphocytes and natural killer T cells. The depletion of these cells is linked to the administration of obinutuzumab, primarily targeting B cells but also affecting T lymphocytes and natural killer T cells.^12^, ^15^

Obinutuzumab, a type of anti‐CD20 monoclonal antibodies, is an immunosuppressive drug that requires special attention to the risk of COVID‐19 pneumonia. According to one report, more than 15% of patients who were infected with COVID‐19 virus within 6 months of receiving anti‐CD20 monoclonal antibodies, including obinutuzumab, experienced relapse of COVID‐19 virus infection.^11^ Furthermore, according to another report, when compared with rituximab, another anti‐CD20 monoclonal antibody, obinutuzumab has been associated with worse clinical outcomes, increased hospitalization rates, and more severe disease progression in patients infected with the omicron variant.^16^ Not only patients on immunosuppressive drugs but also those with untreated or advanced human immunodeficiency virus (HIV) disease are considered to have a persistent COVID‐19 infectivity even months after initial detection,^17^ and their virologic duration extends as long as almost a year.^17^ Considering that depression is the most common mental health condition among individuals with HIV or acquired immune deficiency syndrome (AIDS), with a prevalence as high as 44.9% in one study,^18^ psychiatrists should be aware of the possibility of this persistent COVID‐19 infectivity, which may even occur in psychiatric wards.

Administration of ECT during COVID‐19 infection in patients like our case has been rarely documented^19^ and ECT should be performed after considering its benefits and risks. Given the potential for prolonged infectivity, ECT treatment might be beneficial for patients with COVID‐19 infection and extremely severe depression if appropriate infection control measures are taken, as it often leads to shorter hospitalization and faster recovery as well as a reduced isolation period. Once a cluster occurs in a psychiatric ward, it tends to spread throughout the entire ward. Therefore, when those patients are admitted to a psychiatric ward, careful monitoring of the risk of recurrence and sufficient consideration for infection control measures are necessary to avoid outbreaks and facilitate effective treatment.

## AUTHOR CONTRIBUTIONS

Masaki Nakano, Riko Wakisaka, and Taketo Takata acquired case data. Masaki Nakano, Michitaka Funayama, and Shun Kudo drafted the manuscript. Masaru Mimura, Hiroyuki Uchida, Shin Kuramochi, Akihiro Koreki, Satoyuki Ogino, Takuto Ishida revised the manuscript. All authors read and approved the final manuscript.

## CONFLICT OF INTEREST STATEMENT

The authors declare no conflicts of interest.

## ETHICS APPROVAL STATEMENT

This case report was conducted in accordance with ethical guidelines for case reports of the Japanese Society of Psychiatry and Neurology.

## PATIENT CONSENT STATEMENT

Written informed consent was obtained from the patient for publication of this report and any accompanying images.

## CLINICAL TRIAL REGISTRATION

N/A

## Data Availability

The data of this case report are available from the corresponding author (M.N.) upon request.
